# Assisted suicide within long-term care facilities for older adults: organizational issues and processes experienced by health and social care providers in Switzerland

**DOI:** 10.3389/fpsyt.2025.1537038

**Published:** 2025-03-11

**Authors:** Dolores Angela Castelli Dransart, Elena Pedrazzini Scozzari

**Affiliations:** School of Social Work, HES-SO University of Applied Sciences and Arts Western Switzerland, Fribourg, Switzerland

**Keywords:** assisted suicide (MeSH), assisted death, nursing home, attitudes of professionals, ethical and organizational issues, communication, qualitative study, Switzerland

## Abstract

**Introduction:**

Assisted suicide is still a controversial issue among health and social care providers. They are likely to face challenges in end-of-life care in long-term facilities for older adults, both on organizational and professional levels. Although Swiss professionals are not involved in the final act, they are involved to various extents in the process which leads to the death.

**Methods:**

This qualitative study was carried out in 12 facilities in French-speaking Switzerland, which had been faced with requests for suicide assistance from older adults. A total of 36 professionals (physicians, nurses, nursing assistants, social workers, directors) were interviewed. Data were analyzed according to Grounded Theory principles.

**Results:**

The results uniquely describe how the process unfolds within facilities, from the initial request for suicide assistance to the aftermath of death. This process gives rise to many questions concerning the most appropriate ethical, professional and organizational way to respond to the requests and provide specific support to the requesting person, their family, and staff within the institution. Institutional life and daily operations are significantly impacted. Major adjustments are required to procedures, usual care routines, resource allocation and communication management within the facility. Institutions that set out a clear framework for the way in which the request is handled and provide sufficient and appropriate support for staff are the least disrupted, not only in terms of their operations, but also in terms of cohesion within teams and relationships between care teams and management.Throughout the process, communication within the institution is fundamental to enable staff members to find their bearings and make sense of the situation.

**Discussion:**

Several cross-cutting issues are identified: the need to strike a balance between respecting the procedures laid down by law or the directives of professional associations, and the need to open up spaces for exchange and the construction of meaning for those involved in the process. Further issues include the preparation and training of professionals, and the support provided to them throughout the process. Training and support seem critical to maintaining the continuity and quality of care, motivation and the health of staff.

## 1 Introduction

Given the increasing number of countries that have legalized or decriminalized it, ever more health and social care providers (professionals) are likely to be confronted with requests for assisted dying. Realities, practices, and terminology vary across countries and over time. The administration by a healthcare provider of a lethal substance to terminate the life of a mentally competent patient who has explicitly requested it has been called voluntary euthanasia in some European countries (e.g., the Netherlands and Belgium) or medical aid in dying in Canada. The provision of the lethal substance by a physician has been called physician-assisted suicide in the USA, when this practice was first allowed in Oregon. More recently, the term “assisted dying” has been adopted. It includes voluntary euthanasia, medical aid in dying, and assisted suicide. In assisted suicide, a mentally competent patient takes a lethal dose of a prescribed substance and puts an end to their life.

Assisted dying is still controversial among professionals. The moral and ethical issues of both pros and cons are often discussed (1, 2). In the Quah et al. literature review (3), professionals were in favor of assisted dying in 16 studies, whereas in 23 studies, they opposed it. Some consider assisted dying as pertaining to their professional practice, while for others, it is inconsistent or even incompatible with their role and professional ethos (4, 5). Assisted dying is likely to represent a serious dilemma for professionals (5–7). That is why, in countries where it is permitted, professionals can usually invoke a conscientious objection clause (8, 9).

Some studies have reported higher endorsement of assisted dying if the physician had a strong relationship with the patient (10, 11) or if patients had terminal illnesses (12, 13). Professionals who have previous experience or exposure to assisted dying seem to be more willing to accept and carry it out, most of all if they have received requests in the last 12 months (14).

Some studies have mentioned a discrepancy between professionals’ attitudes and practice in Switzerland, Quebec, or the USA (8, 12): for example, in the Hetzler et al. study (15), 60% of American physicians supported the legalization of physician-assisted suicide, but only 9% said they would carry it out (25% said they might).

Professionals having faced or performed assisted dying have reported feelings of heavy responsibility, emotional burden (16), and moral or professional dilemmas (5). Medical aid in dying in Canada was found to be impactful for nurses (17); the same is true for assisted suicide for other professionals (5). In some studies, practitioners experienced guilt, powerlessness, moral distress, and loneliness (3–5, 16).

Miscommunication in assisted dying is a source of concern for physicians (18). Communication is also a key issue for nurses (19). Physicians are also increasingly facing different and changing interpretations and expectations of good practices with regard to assisted dying (20).

Data on the experiences and impact of assisted dying at the organizational level are very scarce. It is mostly debated whether assisted dying should be considered a healthcare intervention or, as in the literature review of Franke et al. for the prison setting (21), how the legal or ethical criteria should be interpreted or applied. In palliative care settings, assisted dying has given rise to lively debate or controversy. In particular, it has been questioned whether assisted dying can be compatible with the core purpose and philosophy of palliative care and whether it is acceptable or appropriate to accept it within residential palliative care settings (22, 23). The issue of institutional non-participation has mostly been framed in terms of conscience and religious beliefs (24), but other reasons may be relevant such as quality of care and communication both within staff and with patients (25), the institution’s right to self-governance based on capacity and expertise, principled considerations, philanthropic funding implications, or still the possible conflation of palliative care and assisted dying in the public consciousness, among others (24). Some of these reasons have been debated in the public arena and within Swiss institutions as well where the government has no obligation to guarantee the access and where institutions, depending on the canton in which they are located, have the choice of accepting or refusing assisted suicide. According to Hurst and Mauron (26), the Swiss situation is unique because of the normative context shaped not only by legal provisions but also by ethical guidelines developed by professional bodies and the policies of the Right-to-Die associations themselves, on the one hand, and because of the less medicalized nature of assisted suicide, on the other. There are no federal regulations governing assisted suicide. Assisted suicide is decriminalized under certain circumstances. Any person who provides assistance will not be prosecuted if three criteria are fulfilled: (a) the person who wishes to end their life has capacity and (b) can self-administer the fatal substance, i.e., carry out the final act themselves; (c) the person who assists has no self-interested motivations, as defined by Art. 115 of the Swiss Criminal Code (27). In some Swiss French-speaking cantons, assisted suicide is regulated by state laws on Public Health, which apply to long-term care facilities financially supported by the state. In the canton of Vaud, the law enforcement directives (28) of art. 27d of the Law on Public Health (29) lay down the following criteria: the person must be capable of discernment/must have capacity; their desire to die by suicide is persistent; they must be suffering from a serious and incurable illness or its after-effects. If mental disorders or external pressures are suspected, the opinion of an expert psychiatrist must be sought.

Physicians involved in the process must also comply with the Swiss Academy of Medical Sciences guidelines (30). The provision of medical assistance for an assisted suicide must be in accordance with four criteria that partially overlap those of state law: the capacity of the patient; an enduring wish to die, which is well-considered and not due to external pressure; severe suffering, substantiated by an appropriate diagnosis and prognosis; and alternatives must be sought, discussed with, and offered to the patient.

In Switzerland, suicide assistance is not usually provided by professionals in the course of their duties but mostly by volunteers of Right-to-Die associations. EXIT ADMD Romandie (EXIT), a Right-to-Die association that operates in the French-speaking cantons of Switzerland, requires the person to be affiliated to the association, living in Switzerland, over 18 years old, and suffering from an incurable disease, and have unbearable suffering or invalidating polypathologies linked to older age (31).

People aged 65 or above represent 19.3% of the resident population in Switzerland (10.6% are women) (32). Approximately 5% (4.9%) live in long-term care facilities (33).

Median age at death by assisted suicide between 1999 and 2018 was 82 years (78 for men and 85 for women), and over the same period, the number of assisted suicide cases doubled for each 5-year period compared with the preceding period (34). In 2023, assisted suicide accounted for approximately 2.4% of all deaths (33). Persons over 65 years old accounted for 90.7% of all assisted suicide, and women accounted for 59.8% (34). In Switzerland, there is no obligation to report assisted suicides to a central national registry (35). To our knowledge, no data at the federal level are available concerning assisted suicide within long-term care facilities. However, 19.4% of all assisted suicides carried out by French-Speaking EXIT (one of the several Right-to-Die associations that operate in Switzerland) took place in care facilities in 2023 (36).

According to a Canadian study, people residing in an institution are less likely to die by medical aid in dying (6.3% vs. 28.0%) (37).

So far, most of the data in the literature concerning professionals relate to attitudes, moral positions, or emotions experienced in connection with assisted suicide, and is focused mainly on physicians and nurses (16, 38), principally those working in hospital settings or sometimes in palliative care. There is a lack of data on the whole process, from the request to the final act of assisted dying, and from a perspective that considers not only personal experiences but also organizational issues. However, the integration of assisted dying into care settings is likely to raise specific questions about the purpose of the institution, the continuity and quality of care, and the management of this particular type of end-of-life situation in terms of institutional operations. For example, management may need not only to deal with conflicting reactions and attitudes among staff to assisted suicide, but also to provide a framework that allows professionals to adapt individually and collectively to new situations that may challenge their roles and the provision of care within the institution. In addition, to our knowledge, assisted suicide has not been extensively researched in long-term care facilities for older adults. Long-term care facilities may face specific challenges due to the dual nature of their mission: they provide individual healing or care and are a collective living environment where social relationships and interactions are important, span time, and result in proximity of all actors involved. The aim of this article is to describe the processes that took place within long-term care facilities for older adults as experienced by the various professionals involved, to show how a request for assisted dying was managed within the organization and how the management and professionals dealt with the challenges posed by the request.

## 2 Materials and methods

The qualitative study was carried out in long-term care facilities in two French-speaking states, one of which is the canton of Fribourg, which has no legislation on assisted suicide. Institutions there follow guidelines established by the umbrella organization overseeing long-term facilities providing care for older adults. In the canton of Vaud, state regulations govern procedures for assisted suicide in institutions.

A two-step recruitment process was carried out. In the first step, in the absence of public data concerning assisted suicide within long-term care facility for older adults, and given that assisted suicide is a rare event, all residential homes (226) listed in official registers in the two cantons included in the study were invited by a letter to participate provided that they had been confronted with requests for assisted suicide (whether carried through or not). A written reminder was sent out 3 weeks after the first call.

In the second step, professionals working in facilities wherein management answered the call were then recruited on a voluntary basis and through a purposive sampling procedure. The management of these institutions forwarded the invitation flyer and information about the study. All professionals who were involved in the assisted suicide process within the facilities that answered the call were invited to a semi-structured interview. If they agreed to participate, they were contacted by a researcher by e-mail or phone and a convenient appointment was arranged at the workplace. No one dropped out after giving their consent. The face-to-face interview was carried out during working hours at the workplace, in the sole presence of the interviewer. The inclusion criteria were to be over 18 years old, to be able to express themselves in French, and to have been faced with a request for assisted suicide or involved in the process leading to a death by assisted suicide. For each participating institution but one, two to four professionals were interviewed. This allowed us to better understand the process that led to the assisted suicide from multiple points of view, and to situate the requester within it.

The project was reviewed and approved by the ethical commission in charge for the canton of Fribourg (030/13-CER-FR).

Interviewees provided consent both verbally and in writing. Confidentiality was assured and data were processed according to Swiss Federal legislation and Fribourg Cantonal legislation on the protection of personal data. Only the interviewer accessed raw data. Team members accessed anonymized transcriptions.

Interviewees were given the opportunity to receive the interview transcription and a synthesis of the results if they wished. Data presented in this article were collected from 12 long-term care facilities for older adults (5 in the canton of Fribourg and 7 in the canton of Vaud). Among them, six were considered as large institutions (>70 beds), four were considered as average institutions (40–70 beds), and two were considered as small institutions (<40 beds).

The interview guide was elaborated according to scientific literature and themes were identified during the exploratory interviews with stakeholders (one doctor, one officer at the federal Department of Justice, and one member of the board of an umbrella association for long-term care facilities). It was tested with three professionals (one director, one nurse, and one social worker, not included in the sample of the article). The interview guide (see the **Supplementary Material**) covered the following topics:

- the institution’s official position and internal procedures with regard to assisted suicide.
- how the management and the professionals dealt with the assisted suicide request and how the assisted suicide process unfolded within the institution; and.
- the stances (personal and professional) of the professionals with regard to assisted suicide and the reactions and impacts they experienced during or after the assisted suicide or the impacts they observed among colleagues and residents.

Whenever necessary to clarify and deepen understanding, prompts were used. A total of 36 professionals (26 women and 10 men) aged between 24 and 64 were interviewed: 1 physician, 7 directors, 10 head nurses, 6 nurses, 10 nursing assistants or care assistants, and 2 socio-cultural animators. The large majority (26/36) had more than 20 years of experience and 27 professionals had more than 10 years of service in their current position. A total of 23 had training in end-of-life, palliative care or aging, psychology, or gerontology. The interviewees did not know the interviewers prior to the study.

The older people who requested assisted suicide were 11 women and 5 men, aged between 64 and 95. Professionals took care of them for periods ranging from 3 weeks to 7 years prior to the assisted suicide. According to the respondents, six people had multiple pathologies (an accumulation of several physical conditions such as diabetes with amputation, rheumatic diseases, and chronic diseases); five had cancer and five had a heart, respiratory, vascular, or ophthalmologic condition linked to old age. Other issues such as loss of quality of life, depression, and weariness of life were reported by the interviewees.

The interviewers (three women including the two authors, and one man) were trained in social work (one professor with PhD), social sciences (the research assistant with MA), sociology (one professor with PhD), and nursing (one professor with PhD).

Interviews (from 38 to 107 min in length) were audio-taped, transcribed verbatim, and analyzed with ATLAS.Ti (39) and Microsoft Excel software in several stages.

After every interview, a debriefing occurred, involving the interviewer and at least one other member of the research team. Field notes about the location, the interview process, non-verbal information, and interactional aspects were written down by the interviewer. A reflexive journal was kept by each interviewer over the research period and analytical memos were written consistently over the analysis period.

Because of the lack of data and the relative novelty of assisted suicide in long-term care institutions, we chose a grounded theory approach with the aim of developing theoretically relevant insights from a number of case studies and their comparison, i.e., constructing an explanatory scheme from data that integrates various categories (40, 41). Theoretical sensitivity was achieved through immersion in the data by comparison, by exchanges within the research teams, and by theoretical questioning (42). The analysis of empirical material made it possible to map the way in which institutions and professionals responded to requests for assisted suicide and how they dealt with them. The analyses was carried out through constant comparison and identification of relationships between the conceptual categories that emerged from data and that were established and verified throughout the study (43). The grounded theory uses three types of coding: open, axial, and selective (44). A list of descriptive codes (open coding) was established after having identified emerging and recurring topics through repetitive reading of the material. These descriptive codes were then classified into conceptual categories (axial coding: e.g., management of communication and support), from which dimensions and characteristics were extracted, for example, in communication management: open disclosure of assisted suicide/not, modalities of disclosure, content of disclosure, and people to whom the information was disclosed. Thematic and analytical syntheses for each interview were then written. Following this, metacodes (selective coding) were ranked based on the relationships observed between them: this allowed us to raise the level of abstraction and identify core categories (e.g., the various phases of the process and their components see **Table 1**). Regular meetings between team members provided an opportunity to discuss the development of the list of codes, analyze strategies, and group codes thematically and conceptually into categories, as well as to address possible interpretation biases and researchers’ own representations in relation to assisted suicide. DACD and EPS (the authors) coded the interviews and performed the analysis. Codes, categories, and themes were thoroughly discussed within the research team (four people, see Acknowledgments). Saturation was reached when no new themes emerged after 25 interviews (45). For ethical reasons, and in order to honor the motivation of the professionals and not to exclude any of the people who had announced themselves for an interview, we conducted and analyzed 36 interviews.

**Table 1:** Various phases of the process according to some categories of analysis and major challenges faced.

Process/cross-cutting categories	Materialization of the request		Preparation of the request		Enactment of the request		After death	
	PRO	MAN	PRO	MAN	PRO	MAN	PRO	MAN
Information + communication management	Elicit candidate’s point of view	Consideration of request Involve doctors and head nurses	Adapt communication modalities with candidate and family	Decision-making (partially agreed with the candidate) on - Timing of information - Amount of information - Circumstance/setting of information (regular or special meeting)	Adjust communication with the candidate, their family, other residents, EXIT, and authorities	Guidance on information to share/not within the institution	Adjust communication with family and other residents	Instructions regarding information to share and communication modalities
	Forward information to superior	Decide with the candidate on the scope + modalities of information disclosure–official information	Team communication alignment	Clarify responsibilities and tasks related to the process and to the information or communication management.			Communication among professionals about assisted suicide	Manage information among employees: clarify, reframe, answer questions
		Officially start the process and inform employees					Report to management and answer questions from medical examiners and police	Answer questions and cooperate with police and medical examiners
Dealing with moral/professional/emotional or institutional issues	Dealing with surprise and/or helplessness	Establish an organizational framework and/or official stance on assisted suicide (integration or not into institutional purpose)	Choose whether to work on day of death and choose whether to be in contact with candidate	Allow and organize for conscientious objection or authorization to be in the room Adjust work schedules (staff substitution/reinforcement) and room usage (privacy)	Living the experience - Dealing with emotions such as fear, anxiety, satisfaction, or feeling excluded - Watching/facing the process unfold	Reminding institutional stance and professional frameworks Act as guarantor of the process	Find ways of closure.	protect/do not expose staff and residents to medical–legal proceedings (i.e., bringing police officers through less-traveled routes)
	Facing moral and professional challenges Controversies over compatibility of assisted suicide with professional purpose	Facilitate the expression of diverse viewpoints and frame discussions	Deal with emotions, both on a personal and team level	Address controversies and deal with dynamics or potential tensions within teams (Re-)clarify institutional position	Preparation of the body in advance	Regulate employee involvement Provide/supervise additional staff on the day of death	Deal with different emotions associated with death (sadness, unfinished business, satisfaction for peaceful death, etc.)	Frame emotional expression within institution
	Learn about legal and ethical frameworks about assisted suicide		Adjust care for applicant	Set standards and reference points for continuity and quality of care until the time of assisted suicide	Waiting for death	Waiting for death	Preserve the candidate’s room and deal with emotions associated with the medico-legal procedure	Allocate private spaces for medico-legal procedures
		Contacts with EXIT about their assessment of the situation	Assist candidate in preparation of death	(Re-)establish a clear internal organization for division of labor and use of spaces	Follow directions concerning spaces and movements	Organize spaces to avoid contact between EXIT and residents or police and residents	Participation in rituals following medico-legal procedures	Organization of rituals
					Contacts with EXIT’s volunteer	Contacts with EXIT regarding their assistance—monitoring their actions	Take the time to elaborate the experience	Replacement of personnel as and when required
Support	Confrontation with peers and/or peer support	Clarification of the legal/ethical framework, guidelines, or institutional protocols	Informal and formal mutual support (receiving/giving)	Ensure clear and sufficient communication throughout the preparation phase		Allocation of additional internal/external resources: -To care for the applicant and family (to be with them) -To care for the staff involved on the day of the death (working in pairs, meetings, defusing or debriefing).	Mutual informal support (discussions within units)	Provide additional internal/external resources for family and staff for meetings, rituals, supervision, opportunities for venting and collective processing of emotions related to assisted suicide
		Provide/Organize spaces for exchanges Discussions of request in meeting (ordinary or extraordinary)		Allocation of additional internal/external resources:- For daily support of the candidate and their family - For staff: to enable discussion and facilitate collective elaboration of the process				
		Call on internal or external expertise for consideration of request		Determine the level and type of support to be provided on the day of death and beyond				
				Contacts with EXIT to coordinate their intervention				
				Contact with candidate’s family				

PRO, professionals; MAN, management; Candidate, person requesting assistance in suicide.

## 3 Results

In what follows, we present the principal stages of the process that takes place in long-term care facilities for older adults when assisted suicide is requested from the point of view of the interviewees: how the older adult’s request materializes and is formalized, how their request is handled and processed by professionals and management involved in preparing for the assisted suicide, and what happens on the day of death and following the assisted suicide.

### 3.1 Materialization and formalization of the request

Our data highlighted two types of scenario related to how the older person’s request for assisted suicide materializes and is formalized: the first type was a request that occurred in stages. Some older persons, for example, indicated that they had registered with EXIT upon entry to the care facility. Others spoke about it when undergoing their annual care assessment.

“*She always said, right from the moment she arrived, that she was a member of EXIT. And in fact, it was during the summer that she started talking about it again, about her need to move ahead with this undertaking and to put an end to her life. So, after that, all the protocols were followed about having discussions with the people involved*” (9_S_head nurse).

In this first scenario, the older persons initially evoke their wish orally, often to the carers looking after them on a daily basis. When this wish becomes a more concrete project, it is relayed to management or to managing carers either by the persons themselves, or by the intermediary of the carer who relays the information to their supervisor. At this point, management invites the person concerned to formalize their request in writing.

“*And when I received her letter - I said to her ‘Well, Mrs. X, I have received your request, I will respect it, (…) we will go through the procedure, it’s your choice, it’s your right. So I am going to pass it on to the doctor and, well there you are, if that’s your choice*’” (12_D_director).

However, the person making the request must also undertake steps to begin the process with the association EXIT that will provide real-life assistance with the suicide. People who have difficulty with putting their request in writing are often helped to do so by family members. In our sample, concrete assistance from a carer was only provided on one occasion: the professional held the phone handset for the older person, as she was disabled, which enabled her to contact EXIT (in the same way that she aided this person to call her family). In general, both management and staff members refuse to participate materially in putting the request into action, in order to avoid any confusion between the role of the carer and that of the person assisting with suicide, or to avoid being accused of having influenced the person.

In the second type of scenario relating to the materialization and formalization of the request, the request was directly submitted to management, without the older person having shared their thoughts and wishes with those caring for them on a daily basis. As a result, the carers perceived the decision as abrupt and drastic. In two of the situations examined, the ones that occurred the longest time ago, the care facilities and their staff were presented with a *fait accompli*, as the date determined for the assisted suicide was only communicated to them a few days beforehand either by the requesting person or by EXIT. Faced with this situation, the professionals felt helpless.

“*So if I go back over it all now, how it was all set up, it was the rush in which it all happened. Two and a half days, three at a push, between the Monday afternoon at two pm and the Thursday morning at ten am, that was really something that was hard to handle. Because really, we’re not prepared, we don’t know what’s going to happen. We have to deal with a team, an institution, a doctor, a family, a resident, everything. And that, that rush was really hard to handle. The fact that we weren’t able to talk things over with the family, was something I found really hard to handle*” (4_D_head nurse).

The request for assisted suicide resulted in quite a bit of questioning on behalf of the professionals involved, both on a personal and a professional level. Those whose personal values or professional ethos did not align with assisted suicide were those who were the most profoundly affected. In particular, staff members who came from a country where assisted suicide is not allowed, or is not possible for cultural or religious reasons, found it difficult to accept the request and the institution’s involvement in the process. Some of them tried to question the older person’s decision, despite the directives given by most of management of the institutions who, sometimes out of fear of EXIT, asked their employees not to influence the older person and to “remain professional and neutral” (12_D_director).

Once the request is official, the management almost always involves the establishment’s doctor, the person’s own doctor and care managers in its discussions. Together, they become the guarantors of the process or the procedure that will be put in place.

Regardless of whether or not the assessment of the person’s situation and condition was conducted in-house or by EXIT, most institutions took the time to discuss things with the person concerned and to understand what had led them to make such a request. Often, the institutions offered care and pain relief alternatives to the older person.

“*We offered this lady other alternatives: we got the doctor to come, we offered pain relief, which she took. We also offered psychological support. We were there, too, for psychological support, which she took up, that lady. She didn’t say no to everything*” (5_D_head nurse).

### 3.2 Preparation for the day of death

Concrete preparation for assisted suicide generally begins when EXIT communicates its acceptance of the person’s request and continues up until the day of death. Depending on the time frame, the preparation was more or less significant and involved a variable number of professionals, as well as the families.

“*It took nearly six months from the time she began saying: ‘I want to go’, the time for her to talk to the doctor, the time for her daughter to talk to the doctor, to speak with the team, the time for them to call, yeah, it was several months, you know, it didn’t just happen from one day to the next*” (7_E_head nurse).

There are a number of important moments during the preparation period: setting up the accompaniment process for the requesting person, managing information and communication both within and outside the institution, and providing support for care teams and staff, as well as organizational logistics.

#### 3.2.1 Accompanying the requesting person

According to the interviewees, both the management and the staff have invested significant amounts of time and energy in accompanying the person who has requested assisted suicide. In the professionals’ view, the person’s decision was respected, as were their wishes as to how that decision should be implemented, within the limits of what was possible for the institution. For the practitioners involved, this often meant having to think about and find new ways of supporting and accompanying the person, adapted to their particular situation.

According to the interviewees the persons concerned were generally able to choose the date and time of their passing, with one exception: in one situation, the management did not wish the assisted suicide to take place just before the weekend, due to their understaffing.

“*So when, all of a sudden, you have to settle on a date, it’s just … it’s quite a tough moment, huh? (…)I think she wanted to do it on a Friday - and so I said ‘But we can’t’. I said, ‘I, with my team, I have to protect my team, we won’t do that before the weekend, I think there are things relating to it … that need to be done, we also need a bit of time. So we left things at least for a good week. And I think that for us, it allowed to think’. ‘How are we going to do this, to think about how we can make everything go as smoothly as possible?*’” (12_D_head nurse).

Generally, the professionals supported the person concerned in several areas: in dealing with their belongings, in leaving certain objects to third parties, and in organizing where the person was able to say farewell to their loved ones, to staff members, or to other residents (if they had been told what was happening).

#### 3.2.2 Managing and communicating information within the institution

The question of how to manage information relating to the assisted suicide request has always been considered a very delicate one: a great deal of time and energy are dedicated to it.

According to the professionals, the wishes of the person in question were taken into account, both in relation to the information given to staff and that given to other residents. If the person did not wish for their decision to be widely known, discretion was the mode adopted within the institution: only the information necessary for the institution to function normally was passed on internally. Nonetheless, on several occasions, it was the person themselves who informed some of the carers or residents, which went against the desire for discretion they had expressed to and agreed upon with management. The management had different ways of informing their staff. Five directors chose to provide information right throughout the assisted suicide process, for example, when the formal request was submitted to management, when the request was assessed, or when EXIT agreed to provide assistance to the person. Other directors decided to provide information from the moment that EXIT set the date for the assisted suicide.

The scope of people informed also varied: some institutions told the whole staff.

“*[ … ] We told the whole institution, well the care staff first, hey, the people who were involved and so on. And each time, we thought about it and set it up, so that it was a transparent process, from what we do, what we say, how we say it, with what, what do we need to put in place for the team, and there you go, we went ahead step by step like that, because we have procedures, well really a cross-sector meeting every fortnight. So, from that fact, there were things, we also put it in writing for the carers but also for the service personnel, (author’s note, to know), huh*” (12_D_head nurse).

Three institutions elected to only inform the teams involved with the older person on a daily basis when the request was made, but then once the date had been determined, they provided information on a broader level. Finally, some institutions only informed the immediate circle of carers, right up until and including the day of death.

“*So there were several team meetings and when the date had been set, we didn’t tell the team straight away, but [we did] on the Thursday in relation to the following Monday*” (9_E_head nurse).

Generally, it was only the assisted suicide situations that occurred the longest time ago that were hidden to some degree within the institution.

“*In the first case, only the nurse manager knew and the staff watched as the police arrived. It had been done a bit secretively (…) Me, I came along later, but I saw that the staff had been really, really upset by this happening*” (7_E_nurse).

The management and supervisors informed staff during the usual team meetings or sometimes by organizing get-togethers or unplanned meetings.

Regarding the other residents, the information was circulated in various ways. No management spoke openly of the person’s assisted suicide to other people living in the institution before it occurred. There were different reasons for this: either because the institution had agreed with the person that a certain level of discretion was required to avoid effects such as inciting others, shocking people whose beliefs were incompatible with assisted suicide, attracting unwanted media attention, or affecting the reputation of the care facility; or, again, because the person wished themselves to keep it quiet. On one occasion, however, it has happened that the person confided in some of their closer residents, which sometimes makes it difficult for the professionals.

#### 3.2.3 Accompanying staff

Managing information and communication is undoubtedly one of the key strategies when accompanying and supporting staff. In choosing different times to relay information, the management and nurse supervisors helped to ensure regulation at various levels (affective, cognitive, and individual–collective), by allowing the professionals to follow the process and to find their place in it. All the interviewees considered that an assisted suicide that takes place in a collective environment entails significant issues, which is why a great deal of thought was put into it beforehand, with a particular focus on staff. Some directors considered that all staff needed to be supported, while others focused their efforts and the support they offered on staff who were likely to be actually involved in accompanying the person who had requested suicide assistance.

Several institutions chose to provide regular reference points, situating each stage within the internal procedure or in relation to cantonal law, enabling staff to follow the progress of the request and deal with emotions.

“*And always, every time a stage passed, let’s say the doctor had sent the letter, we said to the team, ‘There you go, that’s where we’re at’, and we chatted about it. Afterwards, I can’t remember if it was the psychiatrist from the mobile unit or the psychiatrist who came after to see the lady, he was also there for the team, putting things into perspective, you know, to open the discussion and all. That was something that was really appreciated. The care staff, even if the residential area there is very Protestant, I think that the carers are far and away Catholics in the majority, with other habits and customs too. So I think that there as well, well, they were able to express their emotions, how they were feeling*” (12_D_head nurse).

Support was offered mainly in the form of opportunities to express and perhaps even vent emotions about the real-life situation, both individually and collectively, in sessions. It was almost always the nurse managers who were made responsible for providing support to their colleagues.

“*As a team, we were able to say well, here we are and then say ‘well yes, it’s normal, you have the right to say what you feel, to say that I’m afraid, afraid that it will be too hard for me’ and to be able to ask the questions that some of the carers had, like ‘is the person getting everything that they need?*’” (2_E_nurse).

Sometimes, sessions were organized using external resources (psychiatrists or other resident physicians) to help people to express themselves. Having these spaces to get information and express emotions was deemed very important by the managers in helping to control interactions and internal team functioning, and continuing to ensure their cohesion.

“*I think that we anticipated it all really, we thought about it a lot, we really put everything in place, considering the psychological aspects for everyone: the care team, the family, yeah, the service team, the kitchen staff if necessary, really all the people who are around the person*” (12_D_head nurse).

However, a number of professionals would have liked the discussions to also touch on other fundamental questions and not only the feelings, for example, by addressing the question of whether assisted suicide was compatible with the institution’s mission, or with professional responsibilities or ethics.

#### 3.2.4 Organizational logistics

Once the date for the assisted suicide has been set, the institution has to make the necessary arrangements and prepare the staff so that everything goes as smoothly as possible. This involves the organization of work schedules and spaces.

“*We were able to let the team know the date. And we left people the choice, because some of them were still against the person’s decision and really didn’t accept it on the inside of themselves, in relation to their values, we said to the team that those who didn’t want to work that day could have the day off, we looked at who agreed to it. In any case, we knew that for the other residents we’d have enough people to work, because you still have to look after the others. We left it up to the team. Only one person from the team wanted to be off that day*” (5_D_head nurse).

According to the directors, only a small minority of staff members asked not to be present on the day of assisted suicide. The institutions accepted their request and found replacements for the team, so that they were not confronted by the situation. This was sometimes a delicate operation in the smaller institutions.

The management also reinforced the teams on the day of death, in anticipation of one or even two professionals needing to take care of the older person for longer or manage the situation (final wash, interacting with EXIT, and receiving the police and the medical examiner), and so that time would be available for exchanging within the teams following the event, without anything affecting the routine running of the institution.

“*So we reinforced the numbers on the team so that carer only had that lady to look after and didn’t need to run around to other rooms and so that we had time to have a team meeting. As I said, we had a debriefing afterwards. So that, that’s something you have to organize, because you need time. So we increased the numbers on the team*” (5_D_head nurse).

Management also ensures that the person concerned and their family can enjoy a certain degree of privacy, and that neither staff not directly involved nor other residents are unnecessarily disturbed by the assisted suicide act.

“*On that floor, honestly, we had emptied it out. The activities team had taken a number of the residents to have a meal, those who were mobile. Those who were more disabled went to the dining room, so [in] the whole zone where the assisted suicide was going to happen, I knew we weren’t going to see anyone*” (4_D_head nurse).

Finally, the management had to coordinate with EXIT. According to the interviewees, collaboration with EXIT during this preparatory phase was variable: in some situations, it was seen as positive, i.e., the exchange of information was fluid. In these cases, EXIT planned and indicated when its visits to the older person would occur, keeping the institution informed of the situation, discussing things with the team and explaining what was going to happen and how, or taking into account organizational imperatives in setting the date and time for the assisted suicide (e.g., not setting a date on the weekend). This was greatly appreciated by the institutions and associated professionals, who felt that this type of support was respectful, both of the older person and of the institution and its staff. On the other hand, when the institution or staff were exceptionally presented with a *fait accompli*, collaboration was deemed problematic and difficult to cope with.

“*And so things went very fast (…) and between the moment when the Exit volunteer told us and when it actually happened, there were only about 48 hours … Which was huge and which put a huge amount of pressure on Management, so the institution and on the doctor. And the doctor was confronted with - well, I was too - but the doctor mostly, was faced with completely stupid threats from the EXIT doctor and others, like ‘If you don’t do this, well we’re going to sue you, because everything is settled, it’s all in order’, even though that wasn’t the case. And our thoughts, I’m telling you, that were foremost there, were ‘What happens to this person if we don’t?*’” (11_S_nursing assistant).

These difficulties contributed to mounting tensions within the institution.

In most situations, contacts with EXIT fell somewhere between these two extremes, with a variable level of information exchanged and communication of the selected date with several days’ notice.

### 3.3 The enactment of assisted suicide

On the appointed day, special support for the person concerned was planned for and provided.

The act of assisted suicide took place in the resident’s room within the institution, except in two cases: in one, the death took place in a room intended for family gatherings, as the resident shared their room with another person; and in the second, the resident returned to their home to carry out the act.

Four directors told their staff that they were not allowed to be present during the act, while others allowed it if the person had requested it and the attending carer agreed. Professionals were thus confronted with one of four scenarios: either they were not in the institution at the time of the assisted suicide; or they were in the institution but had no contact with the older person on the assisted suicide day; or they accompanied them until EXIT arrived (washing them, tidying up, and spending the last hours with them) but then left the room; or they were present during the act in the person’s room.

The professionals who had final contact with the person were not automatically selected by their supervisors but could choose whether or not to take on this task, on a voluntary basis. In the majority of cases, they did not wish to be alone in facing the situation. Two professionals were therefore sent to accompany them. The institution therefore had to adjust to this self-designation process and make additional resources available in the days leading up to and on the day of the assisted suicide.

The majority of people interviewed left the room before the person took the lethal substance. Four were present when the substance was administered, with the permission of their managers. One person subsequently regretted having been there.

Generally speaking, all interviewed reported a particular atmosphere on the day of assisted suicide, with the words most often used to describe it being: tense, a heavy atmosphere, difficult to concentrate on the usual tasks, a feeling of time suspended, agitated, and questioning things.

“*Well, it wasn’t always just waiting, because at the same time, we kept doing the rest, we were with the other residents, we talked about other things. But it’s true that when all of a sudden - I can’t remember the time anymore - but let’s say at nine am, that was the planned time for her to drink the substance, I did look at the time and said to myself “But how’s it going, is she going to do it or at the last minute, will she say ‘no, after all, I won’t’?*”“ (9_E_head nurse).

All of them said they felt relieved when “everything was over”. According to them, some deaths were quick (in a few minutes) and trouble-free, while others, those that occurred furthest in the past, were more laborious (over a few hours).

Collaboration with EXIT on the day of assisted suicide was assessed in different ways: some institutions considered it constructive. In these cases, the volunteer introduced themselves to the caregivers, reported when the person had died and talked to the professionals following the death. Other institutions reported that they did not really exchange much with EXIT on the day of death. One institution said that they had not been told that death had occurred:

“*And so I simply asked them, ‘Listen, I’m just asking you, when she dies, to let us know’. And so I chose to stay in the nurse’s station on the floor, five meters away from the room. Tenam, they went into the room, 10.20, 10.30, 11.45, still nothing. We said to ourselves ‘Well, seems like there’s been a problem’ OK. Seems like it’s something that can happen. In the end, I went back down to the ground floor and found myself face to face with a man in uniform [ … ] I said, ‘But wait a minute, no one said anything to me’ ‘Oh yes, we were just called a moment ago’. There was a series of misunderstandings between us and Exit that I found hard to cope with*” (4_D_head nurse).

Most interviewees appreciate the distinction between tasks and roles that exist between the staff on one hand, and EXIT on the other, especially on the day of death. None of them, with one exception, feel that it is their responsibility to provide material assistance with suicide, and therefore do not see assisted suicide as something that could perhaps be integrated for all intents and purposes into the end-of-life care of residents, or as something that the institution should be involved without the assistance of an association.

### 3.4 Following the death

Assisted suicide represents a particular type of end-of-life and death within an institution. Even the professionals who support it the most point to its uniqueness and to the differences in process between assisted suicide and other types of death occurring within the institution. For example, staff cannot carry out a post-mortem wash, prepare the body, or arrange the room (placing flowers) as they usually would. The care providers together with the older person therefore have to anticipate a number of things and “prepare” the person in advance:

“*We got her dressed beforehand. So I washed her, I got her dressed before (…) she was ready. Afterwards, we didn’t touch her again, no. No, we had to leave her in the bed, just laid her down because she was sitting up and was tipping over, just laid her down, we couldn’t touch her after that. Because they had to take photos, the police were taking photos. Because when there’s a suicide, there are photos of the body and so on. (…)It was a different kind of death. Everything was really different and that, it was different too, the preparation*” (5_E_nursing assistant).

In fact, as assisted suicide is classified as an unnatural death, the police are called and the medical examiner comes to carry out checks, to ensure that there has not been any undue external influence. During this time, staff are forbidden from going near the body and from going into the room where the death took place. These forensic procedures often reinforce the feeling of having been excluded from end-of-life care, and of not having been able to accompany the deceased to the end.

The interviewees find the investigation trying, even though they recognize it is necessary. The investigation is the moment when the “cold, hard reality” breaks into the proceedings, despite the best efforts of the police and the medical examiner. Caregivers present in the room at the time of death are questioned by the police, and their status shifts from being observers to witnesses. In some cases, the police also questioned carers who were not present at the time of the act about the deceased’s state of health and the care provided up until the time of death. Some institutions had to produce the files and all documents related to the process that led to the assisted suicide. In certain situations, investigations were opened when the police had doubts about whether the legal criteria for eligibility had been respected.

“*So they (author’s note, the police) went much further than EXIT (author’s note, they checked to make sure…) that she wasn’t depressed, that she was capable of discernment, so that we could not be accused of non-assistance to a person in danger*” (5_E_nurse).

The investigation situations that were most detailed were also the ones that occurred the longest time ago of those in our sample. According to the interviewees, the increase in the number of situations occurring in institutions has been accompanied by a simplification of procedures.

Some directors commented on the delicate position in which they find themselves: in cantons where there is existing legislation, the institutions have to agree to allowing an assisted suicide to take place on their premises. Notwithstanding, they are often only involved peripherally and do not have much influence on the evaluation that EXIT makes. Despite this, they do have to answer to the police and may be implicated in criminal proceedings if the public prosecutor decides to open a case on the basis of the report made by the medical examiner or the police. As a result, some directors would prefer to see the investigation, or at least some State-supervised, formal process of criteria verification, taking place before the death by assisted suicide, rather than as a post-mortem procedure.

Generally, after the assisted suicide, management or supervisors bring together staff who were involved in accompanying the deceased person, to share, to debrief, and sometimes for a moment of contemplation. If the professionals were present at the moment of passing, they share their impressions and accounts of how things went with their colleagues. More rarely, psychiatrists or other external professionals are called upon to meet with teams or staff if they wish it.

Approximately half of those interviewed said they had been greatly affected by the assisted suicide (feelings of confusion, sadness, revolt, dreams, and questioning their professional duties). Those most affected would have liked to receive more support, and for a longer period of time. In fact, with one exception, after debriefing on the day itself, none of the institutions offered any further opportunity to discuss the experience afterwards. The institution whose management offered support a few weeks after the death saw that there was a real need for it among staff:

“*Because we had a little debriefing session a few weeks afterwards with someone specialized, so that the team could let go of it all [ … ] It went well. There were members of other teams who came as well, because they also had things they needed to let go of (…). And it’s true, I didn’t realize it, but there were a lot of people who had dreams, had nightmares, who had recurring ideas, who were asking themselves (author’s note, how that happened), all of that. I had a nursing colleague who dreamed of me, that I was strangling the deceased person in their bed*” (3_D_nurse).

A number of professionals have called for the creation of spaces where they can reflect on their professional mission and ethos, and on the care they can offer in these special situations, beyond organizational management and procedure. Similarly, a need for training in this area was noted on several occasions following the event.

The period after the assisted suicide is also a delicate moment for the institution’s other residents, who often only realize that something unusual has happened in the institution when the police arrive. According to most interviewees, the police arriving in the institution left many with questions and even frightened some of the residents. Management and supervisors find ways of limiting such effects, often bringing police officers through less-frequented ways (back entrances and fire escapes).

Two institutions spoke in detail about how the death had occurred after the event. Ten institutions gave no information at all to other residents, either because the practice within the institution did not provide for disclosure of the cause of death; or to “protect” the person concerned, their family, and the institution’s reputation; or so as not to upset the other residents. However, most professionals in these facilities answered the residents’ questions, some not explicitly mentioning assisted suicide, while others did:

“*Of course it disturbs how the unit works, huh I mean. Of course it disturbs how it works, because there are comings and goings, there’s the police, there’s the medical examiner. Of course it disturbs how the unit works. But we also responded to the residents who asked questions and wanted to know what was happening. We also answered that it was a resident who had chosen to pass away with Exit. We didn’t publicize it in the home that they had passed away with Exit. But for people who asked the question directly, we answered it*” (3_D_director).

In terms of post-mortem rituals, the facilities treat assisted suicide in the same way as other deaths: in their eyes, this is very significant, representing an important moment that allows professionals to “close the circle” and to have the feeling that they have finished accompanying the person. In some institutions, accompanying a person ends with attending the funeral. When that is not possible following an assisted suicide (because the family did not want it), the care teams found it difficult.

## 4 Discussion

In Switzerland, professionals are not actually involved in the final act, since it is the person who ends themselves their life. Similarly, the substance is not provided by the professionals who work in long-term care facilities, but usually by an association for the Right to Die with Dignity. However, management and professionals are involved, in various ways, in the process from the initial request to the aftermath of death.

The description of the process experienced by management and professionals shows that a request for assisted suicide within a long-term care facility is not a trivial event: it generally gives rise to a large number of questions concerning the most appropriate way to respond and follow up as shown also in previous research (10, 46). Similarly to results reported in studies within palliative care, the core purpose of the institution was questioned (22–24). The process that led to the assisted suicide required a whole series of specific support steps for the requesting person and their family, and staff within the institution. In all the situations investigated, institutional life and daily operations were significantly impacted.

The normative context (presence or absence of a law or formal institutional procedure) did not seem to play a major role in the way directors and staff dealt with requests. This may be related to the fact that once assisted suicide has been introduced in the institution, it seems not sufficient to follow a formal or administrative procedure, as assisted suicide involves personal, collective, moral, ethical, and organizational issues that require discussion and elaboration specific to each situation. Our findings suggest that the style of management and the choices made in terms of communication and involvement of staff during the process or still the support provided, dynamically adapted, were critical. In institutions where management set out a clear framework for the way in which the request would be handled and provided sufficient and appropriate support for staff over time, disruptions were less important, not only in terms of operations, but also in terms of cohesion within teams and in relationships between care teams and management. The need of support is frequently mentioned in previous studies (22, 23, 25).

From an institutional point of view, major adjustments were required in procedures, daily operations, and information processing or communication within the institution, as well as in the allocation of resources. For example, additional resources had to be allocated on the day of assisted suicide in order to be able to accompany the person to the point of death as well as ensuring normal functioning and accompaniment of the other residents, or to assist with the medico-legal procedures that follow the death. Lack of resources was sometimes an additional difficulty or barrier in the process for institutions as mentioned in a previous study (46) and some directors raised questions related to equity.

Respondents who had a closer relationship with the person requesting assistance found it easier to endorse the request, in line with previous findings (11, 16).

Communication within the institution throughout the process was fundamental in enabling the various staff to find their bearings and make sense of the situation, as well as to prepare as serenely as possible. These results are in line with previous studies that point to communication as a major issue that can be both a source of difficulty and a support for professionals (16, 46, 47).

By informing staff from the various professions at different times and creating spaces for exchange, discussion and even emotional venting, the managers enabled staff to situate themselves and to be able to anticipate and even integrate the adjustments that assisted suicide introduces into end-of-life care and support within the institution. Staff appreciated the opportunity for interdisciplinary exchange. Interdisciplinarity is considered a facilitating factor in assisted dying situations (46). The amount of time the institution put into organizing the event and accompanying the person requesting assistance was also deemed very important. Situations in which professionals felt they had enough time to accompany the person concerned or prepare for their departure were those that had the fewest negative impacts at both personal and organizational levels.

As the process unfolded, a number of cross-cutting issues emerged: the first was the need to strike a balance between respecting the procedures laid down by law or the directives of professional associations, and the second, to find ways of responding that, while respecting formal procedures, opened up spaces for exchange, emotional management, and the construction of meaning and professional closure for the various people involved in the process. Other issues include the preparation and training of professionals, and the support provided throughout the process, as noted by other authors (8, 16, 22, 46–48). Indeed, some interviewees would have liked more support in the form of more regular exchange forums and prior training related to assisted suicide. The majority of the interviewees also hope that, in the future, assisted suicide will not simply become a matter of procedure and organization, but that discussions on the substance can take place within the teams (49) and the institution, so that both the motivation to care and the health of staff are preserved.

### 4.1 Strengths and limitations

For ethical reasons, institutions and professionals were recruited on a voluntary basis. Therefore, self-selection bias is likely to have occurred and the results, as in most qualitative studies, cannot be generalized. We have no means of knowing if institutions that did not participate in the study were faced with assisted suicide requests or if these situations unfolded differently from the process we described. In addition, the involvement of management in the internal distribution of the call for participation may have contributed to the selection bias, although in our sample, there are divergent opinions on both the acceptability of assisted suicide and the issues of its integration into the institution.

Finally, the variety of professional bodies interviewed and the specificity of the normative Swiss context (no federal law, active role of the Right-to-Die associations), where professionals do not administer the lethal substance and are not primarily in charge of the supervision of the final act, makes it even more difficult to generalize the results. Nevertheless, our results shed light not only on individual reactions and ways of coping with requests for suicide assistance, but also, in a unique way, on how such situations are dealt with collectively and organizationally. This may be of interest for institutions across countries faced with requests for assisted suicide, voluntary euthanasia, or medical aid in dying. Indeed, despite different legal provisions, the management of institutions is likely to face similar challenges such as adjusting internal organization and affairs, allocating specific and extra resources and dealing with employees who have contrasting views on assisted death. Our results are likely to foster new insights in a still understudied field in Switzerland and elsewhere, i.e., the management and collective implications and adjustments related to assisted dying within institutions. The results will enrich knowledge and are likely to guide training for professionals as well as organizational guidelines and management in a Western context where the number of assisted deaths is likely to increase, given demographic trends, population health issues, and evolving social representations about dying.

Future longitudinal research is necessary to investigate how the integration of assisted dying within long-term care facilities and other healthcare facilities impacts professionals’ attitudes, health and social care standards and practices, and the kinds of organizational and professional adjustments that are necessary to provide appropriate care. Observational or even ethnographic research, capable of capturing different perspectives and interactions between various players simultaneously, would also be appropriate.

## 5 Conclusion

Long-term care facilities are not only a place of cure and care, where death occurs, but also a collective environment where living together or community life is as important as somatic care: various groups of people share a great deal of time and, at least to some extent, intention and purpose. According to the interviewees, assisted suicide is a variation of the end-of-life experience that requires major adjustments. Indeed, assisted suicide raises a number of personal, relational, and organizational issues. The scope and the organization of work, the routines and usual professional roles, and the modalities of interaction and communication at the end of life are likely to be disrupted. Our findings highlight some particularly important points when it comes to considering assisted suicide within the institution:

1. The institution should provide a clear and safe framework in which all parties involved can situate themselves in relation to the request for assisted suicide and throughout the process at various levels: ethical, professional, and organizational. That goes beyond the adoption of standardized procedures.
2. Sufficient resources should be provided throughout the process: time and support for candidates and professionals, such as opportunities to vent emotions, discuss the situation from ethical, relational, professional and organizational perspectives, reflect on collaboration, adjust work schedules and routines, and meet with external professionals. Caregivers would be more comfortable and skilled in dealing with such situations, and they would in turn provide tailored support to the person concerned and their loved ones.
3. It is critical to pay proper attention to information and communication management with all parties involved in the process.
4. Specific and multi-faceted training should be consistently provided that goes beyond the provision of information. It should address multiple issues and provide opportunities to develop ethical, professional, and organizational skills (e.g., communication).

Although assisted suicide in institutions is still a controversial issue, and despite sometimes conflicting views on the matter, the perspectives of respondents show that management and professionals have had to develop a common way of responding to the request and a common modus operandi within the institution if they wish to ensure congruence and quality of care to the end of the requestor’s life. Meaningful communication, attention to the needs of professionals, and opportunities for collaboration and mutual support among different professions are likely to facilitate the wellbeing of the staff involved and are critical to addressing the challenges associated with assisted suicide.

## Data Availability

As agreed with the ethic commission and research field partners, raw or deidentified data cannot be shared in any form. Requests to access the datasets should be directed to angela.castelli@hefr.ch.
