# Supplementary material for: Assisted suicide within long-term care facilities for older adults: organizational issues and processes experienced by health and social care providers in Switzerland
Source: Front Psychiatry. 2025 Mar 11;16:1537038. doi: 10.3389/fpsyt.2025.1537038 (PMC11932901; doi:10.3389/fpsyt.2025.1537038)
Supplement: Supplementary file 1 [file Table1.docx]

**Main questions related to the article “Assisted suicide within long-term care facilities for older adults: Organizational issues and processes experienced by health and social care providers in Switzerland”.**

1. Does your institution have a formal position on assisted suicide? If so, which one?
2. Do you have any formalized procedures (protocols, directions, etc.) within the institution or within the team?
3. In your professional life, you have been confronted with requests of assisted suicide.

In practice, what do you do when an older person (resident) makes a request for assisted suicide? (What happens? How?)

-in relation to the person?

-In relation to the team?

-in relation to the institution?

-in relation to the resident's entourage (family, friends)?

-in relation to the other residents?

If you were involved or present in an assisted suicide situation, can you describe what happened step by step? *(if more than one situation, consider the last one please)*

1. What are the professional and organizational implications posed by requests for assisted suicide within your institution?

-for professionals,

-for the other residents,

-for family and friends,

- for institutional operations?

1. When a request for assisted suicide was expressed by a resident what raised the most questions, and from which people?

- How were these questions addressed within the team and the institution ?

- What were the effects or results?

1. In your opinion, what were the consequences of a death by assisted suicide on the family, other residents, professionals or the institutional life?
2. What kind of support did you receive within the institution?
3. In your profession, what ethical guidelines or codes are in force about assisted suicide? What do you think of this ethical framework? Does/did it help you in your practice?
4. In your opinion, is assisted suicide compatible with the general mission/scope of your profession?
5. Generally speaking, what is your personal position on assisted suicide?
6. Going back to the last situation you experienced, what were your reactions when the person told you, or you learned, of their wish to receive assistance in suicide?
7. As a professional, do you feel you have a say in institutional policy on assisted suicide ?
8. What is your opinion about the Right -to Die associations such as Exit and Dignitas?
9. Do you think it is necessary for the State to regulate the practices or issues related to assisted suicide?
